# Associations between water insecurity and reproductive health outcomes among adolescent girls and young women in Sub-Saharan Africa

**DOI:** 10.1371/journal.pgph.0005978

**Published:** 2026-02-12

**Authors:** Alex Bawuah, Deborah O. Okeke-Obayemi, Ruth O. Ejiwale, Sylvester R. Okeke, Sanni Yaya

**Affiliations:** 1 School of Global Studies, Faculty of Social Science, University of Sussex, Brighton, United Kingdom; 2 Department of Counselling and Human Development Studies, University of Ibadan, Ibadan, Nigeria; 3 School of Health Sciences, Massey University, Auckland, New Zealand; 4 Centre for Social Research in Health, UNSW Sydney, Sydney, Australia; 5 Health, Rights and Development, School of Social Sciences, UNSW Sydney, Sydney, Australia; 6 The George Institute for Global Health, Imperial College London, London, United Kingdom; University of Ghana, GHANA

## Abstract

Adolescent girls and young women (AGYW) in sub-Saharan Africa (SSA) face heightened risks of adverse sexual and reproductive health outcomes. Water insecurity may contribute to these vulnerabilities by creating social and environmental stressors that undermine reproductive health and rights, yet this relationship remains understudied. This study examined associations between water insecurity and reproductive health outcomes among AGYW in 29 SSA countries, using recent Demographic and Health Survey data. Modern contraceptive use, early sexual initiation, and early childbirth were assessed as reproductive health indicators, while the type of water source and time required to reach the water source served as measures of water stress. A total of 162,077 AGYW were included. Binary multivariable logistic regression models were used to estimate the associations between water insecurity and reproductive health outcomes. AGYW with access to improved drinking water sources were 16% more likely to use modern contraceptives (AOR = 1.16; 95% CI: 1.10 - 1.22), 7% less likely to report early sexual debut (AOR = 0.93; 95% CI: 0.88 - 0.98), and 6% less likely to have a first birth before age 18 (AOR = 0.94; 95% CI: 0.89 - 0.99) compared with those relying on unimproved sources. Additionally, those who spent 30 minutes or less travelling to their water source were 7% more likely to use modern contraceptives (AOR = 1.07; 95% CI: 1.02 - 1.13) and were slightly less likely to initiate sexual activity before age 15 (AOR = 0.98; 95% CI: 0.92 - 1.03). These findings suggest that water insecurity may play a meaningful role in shaping AGYW’s reproductive health in SSA. Consequently, addressing water insecurity and related stressors could support improved reproductive health outcomes and strengthen efforts to protect the well-being and rights of AGYW.

## Introduction

Water insecurity – inadequate or inequitable access to clean, safe and affordable water for drinking, cooking, sanitation and hygiene [1] – constitutes a major public health concern in sub-Saharan Africa (SSA), impacting a wide range of nutrition, hygiene, mental health, and social outcomes [2–7]. Evidence shows that women are disproportionately impacted in relation to the burden of time and effort committed to collecting water in settings where there is water insecurity [8–11]. Accessing water involves substantial stress and could require long-distance travel, most times on foot, and through difficult terrains [8,10], which have implications for exposure to physical, psychological and sexual violence [8,10,12–17]. Adolescent girls and young women (AGYW) constitute a key population in vulnerabilities relating to water insecurity due to intersecting susceptibility arising from gender and sociocultural norms, as well as threats to reproductive health and rights, including menstrual hygiene [18–21].

Water insecurity could impact reproductive health choices and outcomes, including sexual violence and sexual coercion or inducement [13,14,22,23]. Water insecurity could trigger and/or reinforce transactional sex as the need for water – a basic necessity for survival – can be transacted for sex [23,24]. Evidence indicates that transactional sex is associated with sexually transmissible infections (STIs) [25,26] and unplanned pregnancies [27,28]. The public and reproductive health threat of water insecurity could be more pronounced in rural areas and urban slums. This is because higher levels of poverty and lack of infrastructure in these settings make them more vulnerable to water insecurity [6,9,29–31]. This acute water insecurity could intersect with low or total lack of reproductive autonomy, thereby further exposing AGYW in rural areas and urban slums to poorer reproductive health outcomes.

Generally, water insecurity has been exacerbated by droughts due to climate change, especially in arid and semi-arid areas of SSA [15]. This water insecurity may compel AGYW to spend more travel distance and time, and in the process become exposed to sexual violence, coercion or inducement, which has implications for reproductive health outcomes [8,10,12–17]. The impact of water insecurity is likely to extend beyond adolescence to long-term psychosocial and behavioral consequences. AGYW who experience water insecurity and related social stressors could internalize and embrace potential maladjusted coping tactics that could be used in dealing with situations such as uneven gendered burden in sourcing for water, low school attendance or even a total drop-out, transactional sex, early marriage, sexual coercion/violence. These *coping* or survival measures, when internalized and normalized, not only undermine well-being but also extend inequality and vulnerability patterns that prevent reaching national and global development goals around gender equality and population health.

Despite a potential link between water insecurity and adverse reproductive health outcomes, this area of scholarship is still largely understudied. A study by Shimamura et al. [32] found that increased access to water led to significant increases in school attendance and education/school attendance is protective against adverse health outcomes such as unplanned pregnancies [33,34], and transmission of blood-borne viruses (BBVs) and STIs [35,36]. In the same vein, even at adulthood, education is also protective against adverse reproductive health outcomes and increased contraceptives use [37,38]. Considering the dearth of scholarship on the link between water insecurity and reproductive health outcomes, we analysed Demographic Health Surveys (DHS) data in SSA countries to investigate this relationship to inform health, rights and development strategies.

## Methods

### Data source

The study utilised recent data from the DHS of 29 countries in SSA including Angola (2015–2016), Benin (2017–2018), Burkina Faso (2021), Burundi (2016–2017), Cameroon (2018), Chad (2014–2015), Côte d’Ivoire (2021), Ethiopia (2016), Gabon (2019–2020), Gambia (2019–2020), Ghana (2022), Guinea (2018), Lesotho (2023–2024), Liberia (2019–2020), Madagascar (2021), Malawi (2015–2016), Mali (2018), Mauritania (2019–2021), Mozambique (2022–2023), Nigeria (2018), Rwanda (2019–2020), Senegal (2023), Sierra Leone (2019), South Africa (2016), Togo (2013–2014), Tanzania (2022), Uganda (2016), Zambia (2018), and Zimbabwe (2015).

The DHS is a nationally representative survey conducted in more than 85 low- and middle-income countries, adhering to a standardised protocol and consistent terminology across all participating nations [39]. It uses a structured questionnaire to collect data on a wide range of health indicators, including maternal and child health, fertility, use of family planning, morbidity, and mortality [39]. The DHS adopts a two-stage sampling strategy: first, enumeration areas are selected based on each country’s sampling frame; then, households are randomly chosen within each selected area. Comprehensive details on the sampling design and data collection procedures are provided by Aliaga and Ren [40].

The study employed the women’s dataset (IR file) from the DHS. The total sample consisted of 435,633 women aged 15–49, drawn from the pooled sample. For this study, the sample is limited to AGYW aged 15 – 24 years. Thus, the sample size for this study is 162,077 AGYW after cleaning the data and dropping missing observations (this was done for observations with missing data on primary independent and dependent variables using listwise deletion).

### Variables

#### Outcome variables.

The study used three outcome variables: (i) modern contraceptive use, (ii) early sexual initiation, and (iii) early childbirth. These variables were selected because they are key and widely used indicators of AGYW’s reproductive health in SSA [41,42]. Modern contraceptive use reflects young women’s ability to prevent unintended pregnancies and exercise reproductive autonomy [43]. Early sexual initiation captures the timing of sexual debut, which is associated with increased risks of unintended pregnancy and sexually transmitted infections [44,45]. Early childbirth represents a culmination of reproductive health disadvantages and has far-reaching consequences for young women’s health, education, and economic opportunities [46,47]. While other reproductive health indicators (e.g., unmet need for contraception or antenatal care use) are also relevant, these three outcomes most directly capture pathways through which water stress may influence reproductive health, such as time burdens, opportunity costs, and household vulnerability.

Modern contraceptive use was assessed using the DHS variable “v313”, which records the method of contraception currently employed by the respondent. This is originally a categorical variable with four response categories: (i) no method, (ii) folkloric method, (iii) traditional method, and (iv) modern method. For this study, this variable was recoded into a binary outcome: 1 = using any modern method, and 0 = not using a modern method (i.e., no method, folkloric method, and traditional method). Respondents who had never had sex were excluded from this analysis.

Early sexual initiation was assessed using the DHS variable “v531”, which records the age at first sexual intercourse of the respondents. This is originally a continuous variable (age in years). We created a binary variable (from the original variable) coded as 1 = had first sexual intercourse before age 15, and 0 = had first sexual intercourse from the age of 15 onwards. Respondents who had not had any sexual intercourse were excluded from this analysis.

Early childbirth was measured using the DHS variable “v212”, which records the age of the respondent at first birth. This is originally a continuous variable (age in years). We created a binary variable (from the original variable) coded as 1 = first birth occurred before the age of 18, and 0 = first birth occurred from the age of 18 onwards. Respondents who had not given birth were excluded from this analysis.

#### Independent variables.

There were two independent variables of interest: (i) type of water source and (ii) time to water source. The type of water source was measured using the DHS variable “v113”, which records the source of drinking water. This is originally a categorical variable. Following the DHS guide [48], we created a binary variable coded as 1 = improved source (e.g., piped, borehole) and 0 = unimproved source (e.g., surface water, unprotected well).

The time to the water source was measured using the DHS variable “v115”, which records the time to get to the water source. This is originally a continuous variable (minutes to water source). We created a binary variable (from the original variable) coded as 1 = ≤30 minutes and 0 = more than 30 minutes. This cutoff aligns with the WHO/UNICEF Joint Monitoring Programme definition of basic access to water services [49] and has been widely used in prior studies [50,51].

To further explore how combined dimensions of water stress relate to reproductive health, we created a water-stress profile by combining drinking-water source (improved vs. unimproved) and time to source (≤30 minutes vs > 30 minutes); (1) Low stress (improved source and ≤30 minutes to water source); (2) Moderate stress - source (unimproved source and ≤30 minutes to water source); (3) Moderate stress - time (improved source and >30 minutes to water source); and (4) Severe stress (unimproved source and >30 minutes to water source).

The following variables are included as covariates: age (categorical: 15–19, 20–24), education (categorical: no education, primary, secondary, higher), wealth (categorical: poorest, poorer, middle, richer, richest), residence (binary: urban, rural), frequency of reading newspapers (categorical: not at all, less than once a week, at least once a week, almost every day), frequency of listening to radio (categorical: not at all, less than once a week, at least once a week, almost every day), frequency of watching television (categorical: not at all, less than once a week, at least once a week, almost every day), employment status (binary: employed, unemployed) and health insurance status (binary: insured, uninsured). These variables were selected based on their significant relationship with the outcome variables in the literature [52–56] as well as their availability in the DHS dataset.

### Data analysis

The data were analysed with STATA version 18. Descriptive statistics were used to describe the sample. A binary multivariable logistic regression model was employed to assess the association between water source, time to water source and reproductive health among adolescents and young women in SSA [57]. We also applied a binary multivariable logistic regression model to examine associations between the water-stress profiles and reproductive health outcomes [57]. The survey design (sample weights, strata and primary sampling units) was taken into account in all the regression analyses (the main and secondary analyses) using the “svy” command following the DHS guide [39].

### Ethical consideration

This study did not require additional ethical approval as it is based on publicly available, de-identified secondary data from the Demographic and Health Surveys (DHS) Program. The original DHS data collection protocols were reviewed and approved by the Institutional Review Board (IRB) of ICF International, as well as by national ethics committees and relevant authorities in each participating country. Informed consent was obtained from all participants at the time of the original data collection; this consent was written. The DHS Program strictly adheres to ethical guidelines set by the U.S. Department of Health and Human Services to ensure the protection of human subjects and the confidentiality of respondents. Further details on DHS ethical standards and data access policies are available at: http://goo.gl/ny8T6X.

## Results

### Descriptive summary of the sample

**Fig 1:**
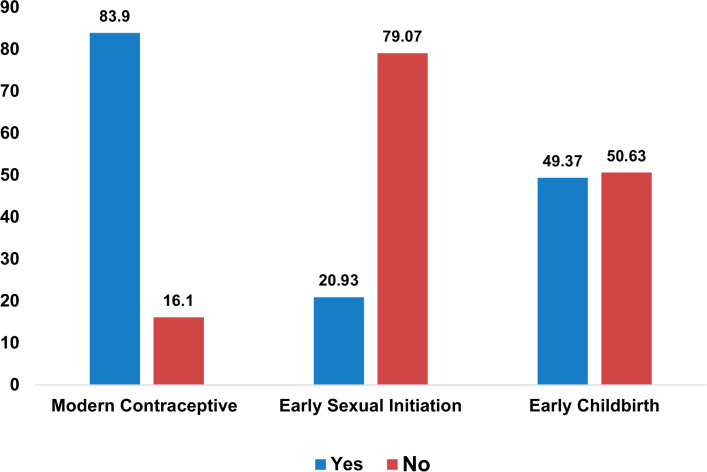
Reproductive health among AGYW in SSA.

Fig 1 illustrates the distribution of reproductive health among AGYW in SSA. It shows that 83.90% of them use modern contraceptives, 20.93% had their first sexual intercourse before age 15, and 49.37% gave birth before the age of 18.

Table 1 presents the results of the descriptive summary of the sample. It shows that most of the AGYW get their drinking water from improved sources (77.56%). Likewise, the majority spend ≤30 minutes travelling to their water source (82.96%). Furthermore, a higher proportion of them are between 15 and 19 years (54.61%), have secondary education (46.38%), are in the richest wealth category (22.18%), live in rural areas (59.69%), currently unemployed (58.74%), have no health insurance (90.06%) do not read newspapers (82.28%), do not listen to the radio (48.56%), and do not watch television (52.87%).

**Table 1:** Descriptive summary of study sample.

Variables	Total sample N = 162,077	Modern contraceptive N = 26,088	Early Sexual Initiation N = 20,734	Early Childbirth N = 31,587
	N (%)	N (%)	N (%)	N (%)
**Sources of drinking water**				
Unimproved sources	36,375 (22.44)	5,013 (19.22)	10,248 (32.44)	6,890 (33.23)
Improved sources	125,702 (77.56)	21,075 (80.78)	21,339 (67.56)	13,844 (66.77)
**Time to source of drinking water**				
> 30 minutes	27,625 (17.04)	4,275 (16.39)	6,174 (19.55)	4,102 (19.78)
≤ 30 minutes	134,452 (82.96)	21,813 (83.61)	25,413 (80.45)	16,632 (80.22)
**Age**				
15–19	88,509 (54.61)	8,205 (31.45)	11,847 (37.51)	9,838 (47.45)
20–24	73,568 (45.39)	17,883 (68.55)	19,740 (62.49)	10,896 (52.55)
**Education level**				
No education	30,900 (19.07)	2,804 (10.75)	10,394 (32.91)	6,676 (32.2)
Primary	50,677 (31.27)	8,880 (34.04)	12,649 (40.04)	8,260 (39.84)
Secondary	75,164 (46.38)	13,233 (50.72)	8,414 (26.64)	5,656 (27.28)
Higher	5,336 (3.29)	1,171 (4.49)	130 (0.41)	142 (0.68)
**Wealth**				
Poorest	30,130 (18.59)	4,041 (15.49)	8,619 (27.29)	5,614 (27.08)
Poorer	30,816 (19.01)	4,824 (18.49)	7,938 (25.13)	5,169 (24.93)
Middle	32,378 (19.98)	5,397 (20.69)	6,769 (21.43)	4,401 (21.23)
Richer	32,801 (20.24)	5,963 (22.86)	5,115 (16.19)	3,330 (16.06)
Richest	35,952 (22.18)	5,863 (22.47)	3,146 (9.96)	2,220 (10.71)
**Residence**				
Urban	65,332 (40.31)	10,870 (41.67)	8,979 (28.43)	6,091 (29.38)
Rural	96,745 (59.69)	15,218 (58.33)	22,608 (71.57)	14,643 (70.62)
**Frequency of reading newspaper**				
Not at all	133,359 (82.28)	20,782 (79.66)	28,633 (90.65)	18,751 (90.44)
Less than once a week	17,593 (10.85)	3,307 (12.68)	1,880 (5.95)	1,213 (5.85)
At least once a week	10,673 (6.59)	1,919 (7.36)	1,023 (3.24)	727 (3.51)
Almost every day	452 (0.28)	80 (0.31)	51 (0.16)	43 (0.21)
**Frequency of listening to radio**				
Not at all	78,697 (48.56)	11,414 (43.75)	17,490 (55.37)	11,848 (57.14)
Less than once a week	33,816 (20.86)	5,600 (21.47)	5,816 (18.41)	3,710 (17.89)
At least once a week	46,861 (28.91)	8,603 (32.98)	7,679 (24.31)	4,760 (22.96)
Almost every day	2,703 (1.67)	471 (1.81)	602 (1.91)	416 (2.01)
**Frequency of watching television**				
Not at all	85,692 (52.87)	13,782 (52.83)	20,941 (66.3)	13,696 (66.06)
Less than once a week	23,721 (14.64)	3,672 (14.08)	3,768 (11.93)	2,474 (11.93)
At least once a week	47,940 (29.58)	7,789 (29.86)	6,013 (19.04)	3,954 (19.07)
Almost every day	4,724 (2.91)	845 (3.24)	865 (2.74)	610 (2.94)
**Currently working**				
No	95,197 (58.74)	12,895 (49.43)	15,531 (49.17)	10,794 (52.06)
Yes	66,880 (41.26)	13,193 (50.57)	16,056 (50.83)	9,940 (47.94)
**Has Insurance**				
No	145,972 (90.06)	23,596 (90.45)	30,064 (95.18)	19,450 (93.81)
Yes	16,105 (9.94)	2,492 (9.55)	1,523 (4.82)	1,284 (6.19)

The results further indicate that most of the AGYW who get drinking water from improved sources use modern contraceptives (80.78%), had their first sexual intercourse before age 15 (67.56%), and gave birth before the age of 18 (66.77%). Similarly, among women who spend ≤30 minutes travelling to their water source, the majority use modern contraceptives (83.61%), had their first sexual intercourse before age 15 (80.45%), and gave birth before the age of 18 (80.22%).

Additionally, a higher proportion the AGYW who use modern contraceptives have secondary education (46.38%), whereas those who had their first sexual intercourse before age 15 (40.04%) and those who gave birth before age 18 (39.84%) have primary education. Also, a higher proportion the women who use modern contraceptives are in the richest wealth category (22.47%), whereas those who had their first sexual intercourse before age 15 (27.29%) and those who gave birth before age 18 (27.08%) are in the poorest wealth category.

### Association between water source, time to water source, and reproductive health among AGYW in SSA

Table 2 presents findings from the multivariable logistic regression model on the association between water source, time to water source and reproductive health among AGYW in SSA.

**Table 2:** Multivariable logistic regression on the association between water source, time to water source and reproductive health among AGYW in SSA.

Variables	Modern Contraceptives	Early Sexual Initiation	Early Childbirth
	AOR (95%CI)	AOR (95%CI)	AOR (95%CI)
**Sources of drinking water**			
Unimproved sources	Ref	Ref	Ref
Improved sources	1.16 (1.10–1.22)***	0.93 (0.88–0.98)*	0.94 (0.89–0.99)*
**Time to source of drinking water**			
> 30 minutes	Ref	Ref	Ref
≤ 30 minutes	1.07 (1.02–1.13)**	0.98 (0.92–1.03)	0.99 (0.93–1.04)
**Age**			
15–19	Ref	Ref	Ref
20–24	3.31 (3.18–3.44)***	0.64 (0.61–0.66)***	0.22 (0.21–0.23)***
**Education level**			
No education	Ref	Ref	Ref
Primary	1.69 (1.58–1.80)***	0.89 (0.84–0.94)***	0.89 (0.84–0.94)***
Secondary	1.79 (1.67–1.91)***	0.45 (0.42–0.48)***	0.46 (0.43–0.49)***
Higher	1.64 (1.46–1.85)***	0.17 (0.13–0.21)***	0.18 (0.13–0.23)***
**Wealth**			
Poorest	Ref	Ref	Ref
Poorer	1.12 (1.06–1.19)***	0.92 (0.87–0.98)**	0.97 (0.91–1.03)
Middle	1.17 (1.10–1.25)***	0.88 (0.83–0.94)***	0.96 (0.90–1.02)
Richer	1.11 (1.04–1.19)**	0.80 (0.74–0.87)***	0.87 (0.81–0.94)***
Richest	0.86 (0.79–0.94)***	0.67 (0.60–0.74)***	0.82 (0.74–0.90)***
**Residence**			
Urban	Ref	Ref	Ref
Rural	0.93 (0.88–0.99)*	1.04 (0.97–1.11)	1.00 (0.94–1.06)
**Frequency of reading newspaper**			
Not at all	Ref	Ref	Ref
Less than once a week	0.97 (0.91–1.03)	0.92 (0.84–1.00)*	0.91 (0.84–0.99)*
At least once a week	0.94 (0.87–1.01)	0.92 (0.81–1.04)	0.84 (0.75–0.94)**
Almost every day	1.18 (0.83–1.66)	0.59 (0.38–0.90)*	0.47 (0.31–0.72)***
**Frequency of listening to radio**			
Not at all	Ref	Ref	Ref
Less than once a week	1.13 (1.07–1.19)***	0.93 (0.88–0.98)**	0.98 (0.92–1.04)
At least once a week	1.23 (1.17–1.29)***	0.90 (0.85–0.95)***	0.94 (0.89–0.99)*
Almost every day	1.10 (0.95–1.29)	1.06 (0.90–1.24)	1.00 (0.84–1.19)
**Frequency of watching television**			
Not at all	Ref	Ref	Ref
Less than once a week	1.07 (1.01–1.13)*	0.98 (0.92–1.05)	1.02 (0.95–1.10)
At least once a week	1.10 (1.04–1.17)***	0.95 (0.89–1.02)	1.00 (0.93–1.07)
Almost every day	1.39 (1.19–1.62)***	1.07 (0.91–1.27)	1.20 (1.01–1.43)*
**Currently working**			
No	Ref	Ref	Ref
Yes	1.38 (1.33–1.44)***	1.00 (0.96–1.05)	1.09 (1.05–1.15)***
**Has Insurance**			
No	Ref	Ref	Ref
Yes	1.01 (0.91–1.12)	1.05 (0.92–1.20)	0.94 (0.81–1.08)
**Country Fixed Effect**	Yes	Yes	Yes
**Constant**	0.02 (0.02–0.03)***	1.06 (0.92–1.23)	7.35 (6.38–8.47)***

**Note:** AOR = Adjusted Odds Ratio; 95% confidence interval (CI) in parentheses; Ref = Reference group. *** p < 0.001, ** p < 0.01, * p < 0.05

The results show that AGYW who obtained their drinking water from improved sources are 16% more likely to use modern contraceptives compared to those who get it from unimproved sources (AOR = 1.16; 95% CI: 1.10 - 1.22). Also, they were 7% less likely to have sexual intercourse before age 15 (AOR = 0.93; 95% CI: 0.88 - 0.98). Likewise, they were 6% less likely to give birth before they turn 18 years (AOR = 0.94; 95% CI: 0.89 - 0.99).

Furthermore, those who spend ≤30 minutes travelling to their water source were 7% more likely to use modern contraceptives than those who travel for more than 30 minutes (AOR = 1.07; 95% CI: 1.02 - 1.13). Also, they were less likely to have sexual intercourse before age 15 (AOR = 0.98; 95% CI: 0.92 - 1.03) or give birth before they turned 18 years (AOR = 0.99; 95% CI: 0.93 - 1.04) – the latter results are statistically insignificant.

The results also show that those who are between 20 and 24 years were more likely to use modern contraceptives (AOR = 3.31; 95% CI: 3.18 - 3.44) compared to those between 15 and 19 years. Also, they were less likely to have initiated sexual intercourse before age 15 (AOR = 0.64; 95% CI: 0.61 - 0.66) and to have given birth before age 18 (AOR = 0.22; 95% CI: 0.21 - 0.23).

Moreover, AGYW with a higher education were more likely to use modern contraceptives (AOR = 1.64; 95% CI: 1.46 - 1.85) than those with no education. Also, they were less likely to have initiated sexual intercourse before age 15 (AOR = 0.17; 95% CI: 0.13 - 0.21) and to have given birth before age 18 (AOR = 0.18; 95% CI: 0.13 - 0.23).

Additionally, AGYW in the richest wealth category were less likely to use modern contraceptives (AOR = 0.86; 95% CI: 0.79 - 0.94), to have initiated sexual intercourse before age 15 (AOR = 0.67; 95% CI: 0.60 - 0.74), and to have given birth before age 18 (AOR = 0.82; 95% CI: 0.74 - 0.90), compared to those in the poorest category.

Also, AGYW who were currently employed were more likely to use modern contraceptives (AOR = 1.38; 95% CI: (1.33 - 1.44) and to have given birth before age 18 (AOR = 1.09; 95% CI: 1.05 - 1.15) compared to those who were unemployed.

### Association between water stress profiles and reproductive health outcomes among AGYW women in SSA

The water stress profile analysis revealed that 66.36% of AGYW experienced low water stress, 16.59% experienced moderate stress due to unimproved water sources, 11.19% experienced moderate stress due to the time to water sources, and 5.85% experienced severe water stress.

Table 3 presents findings from the multivariable logistic regression model on the association between the water stress profile and reproductive health among adolescent girls and young women.

**Table 3:** Multivariable logistic regression on the association between the water stress profile and reproductive health among AGYW in SSA.

Variables	Modern Contraceptives	Early Sexual Initiation	Early Childbirth
	AOR (95%CI)	AOR (95%CI)	AOR (95%CI)
**Water stress profile**			
Low stress	Ref	Ref	Ref
Moderate stress - source	0.88 (0.83–0.93)***	1.07 (1.01–1.13)*	1.05 (0.99–1.11)
Moderate stress - time	0.95 (0.90–1.01)	1.02 (0.95–1.09)	1.00 (0.93–1.07)
Severe stress	0.77 (0.70–0.84)***	1.11 (1.02–1.20)*	1.10 (1.01–1.20)*
**Age**			
15–19	Ref	Ref	Ref
20–24	3.31 (3.18–3.44)***	0.64 (0.61–0.66)***	0.22 (0.21–0.23)***
**Education level**			
No education	Ref	Ref	Ref
Primary	1.69 (1.58–1.80)***	0.89 (0.84–0.94)***	0.89 (0.84–0.94)***
Secondary	1.79 (1.66–1.91)***	0.45 (0.42–0.48)***	0.46 (0.43–0.49)***
Higher	1.65 (1.46–1.85)***	0.17 (0.13–0.21)***	0.18 (0.13–0.23)***
**Wealth**			
Poorest	Ref	Ref	Ref
Poorer	1.12 (1.06–1.19)***	0.92 (0.87–0.98)**	0.97 (0.91–1.03)
Middle	1.17 (1.10–1.25)***	0.88 (0.83–0.94)***	0.96 (0.90–1.02)
Richer	1.11 (1.04–1.20)**	0.80 (0.74–0.87)***	0.87 (0.81–0.94)***
Richest	0.86 (0.79–0.94)***	0.67 (0.60–0.74)***	0.81 (0.74–0.90)***
**Residence**			
Urban	Ref	Ref	Ref
Rural	0.93 (0.88–0.99)*	1.04 (0.97–1.11)	1.00 (0.94–1.06)
**Frequency of reading newspaper**			
Not at all	Ref	Ref	Ref
Less than once a week	0.97 (0.91–1.03)	0.92 (0.84–1.00)*	0.91 (0.84–0.99)*
At least once a week	0.94 (0.87–1.01)	0.92 (0.81–1.04)	0.84 (0.75–0.94)**
Almost every day	1.18 (0.83–1.66)	0.59 (0.38–0.90)*	0.47 (0.31–0.72)***
**Frequency of listening to radio**			
Not at all	Ref	Ref	Ref
Less than once a week	1.13 (1.07–1.19)***	0.93 (0.88–0.98)**	0.98 (0.92–1.04)
At least once a week	1.23 (1.17–1.29)***	0.90 (0.86–0.95)***	0.94 (0.89–0.99)*
Almost every day	1.10 (0.94–1.28)	1.06 (0.90–1.24)	1.00 (0.84–1.19)
**Frequency of watching television**			
Not at all	Ref	Ref	Ref
Less than once a week	1.07 (1.01–1.13)*	0.98 (0.92–1.05)	1.02 (0.95–1.10)
At least once a week	1.10 (1.04–1.17)***	0.95 (0.89–1.02)	1.00 (0.93–1.07)
Almost every day	1.39 (1.20–1.62)***	1.07 (0.91–1.27)	1.20 (1.01–1.43)*
**Currently working**			
No	Ref	Ref	Ref
Yes	1.38 (1.33–1.44)***	1.00 (0.96–1.05)	1.09 (1.05–1.15)***
**Has Insurance**			
No	Ref	Ref	Ref
Yes	1.01 (0.91–1.12)	1.05 (0.92–1.20)	0.94 (0.81–1.08)
**Country Fixed Effect**	Yes	Yes	Yes
Constant	0.03 (0.02–0.03)***	0.97 (0.84–1.12)	6.84 (5.96–7.84)***

**Note:** AOR = Adjusted Odds Ratio; 95% confidence interval (CI) in parentheses; Ref = Reference group. *** p < 0.001, ** p < 0.01, * p < 0.05

For modern contraceptive use, the results revealed that AGYW who experienced moderate stress due to an unimproved water source had 12% lower odds of using modern contraception compared with those in the low stress group (AOR = 0.88, 95% CI: 0.83 - 0.93). The association was stronger for those in severe stress, who had 23% lower odds of modern contraceptive use (AOR = 0.77, 95% CI: 0.70 - 0.84).

For early sexual initiation, those who experienced moderate stress due to unimproved water source were 7% more likely to have sexual intercourse before age 15 (AOR = 1.07, 95% CI: 1.01 - 1.13). Similarly, those who experienced severe stress were 11% were more likely to have sexual intercourse before age 15 (AOR = 1.11, 95% CI: 1.02 - 1.20).

For early childbirth, the results show that those who experienced severe water stress had 10% higher odds of early childbirth (AOR = 1.10, 95% CI: 1.01 - 1.20) compared to those with low stress.

## Discussion

Overall, our findings suggest that water security is protective and could improve reproductive health outcomes of AGYW in SSA. We found that having access to an improved water supply and low travel time to water source were associated with better reproductive health outcomes measured using three parameters – delayed sexual debut, use of modern contraceptives, and not becoming primiparous before 18 years. These results in support of evidence linking water security to improved women’s health and wellbeing [4,6,8,32] suggest that improved access to water may be protective against adverse reproductive health outcomes for AGYW directly and indirectly. Directly, access to water could decrease exposures to vulnerabilities leading to transactional sex and sexual violence [13,14,22,24,58]. Indirectly, access to water reduces school absenteeism attributable to time spent hunting for water [7,32]. Since schooling is protective against adverse health outcomes such as unplanned pregnancies and early marriages [33,34], and transmission of BBVs/STIs [35,36], spending less time hunting water implies spending greater time at school. Also, schooling and education has the tendency of improving AGYW reproductive autonomy [59,60] thereby reducing their vulnerability to sexual exploitation and coercion.

Our results investigating the association between age and reproductive health outcomes suggest that younger AGYW aged 15–19 years, when compared with their older counterparts aged 20–24 years, may be more vulnerable to adverse reproductive health outcomes. This is because adolescent girls (aged 15–19) are less likely than young adults (aged 20–24) to use modern contraceptives, and more likely to report debuting sex before 15 years and become primiparous before 18 years. This could be attributable to the fact that young adults, compared to adolescents, are more experienced, might have spent a higher number of years at school and therefore are more knowledgeable to exercise reproductive autonomy and are more confident to negotiate and make informed sex-related decisions. This finding supports the results of previous studies indicating that older adolescents are more likely to demonstrate higher reproductive health knowledge and autonomy, partly because of higher exposure to education, which improves agency [59–61]. Thus, equitable intervention may need to focus greatly on adolescent girls (15–19 years), compared to young adults (20–24 years), because of the higher vulnerability adolescents may experience, compared to young adults.

In the same vein, our result reinforces the protective role of education and schooling on AGYW reproductive health and rights. We found that AGYW with a higher level of formal education were more likely to report using modern contraceptives as well as less likely to report early sexual debut and becoming primiparous before 18 years old. The protective role of education as a social tool for socio-economic development and attaining health and overall development outcomes cannot be overemphasised. Even though power imbalances and other factors may likely drive sexual exploitation in schools, generally, schooling keeps AGYW safe from sexual exploitation and provides opportunities for acquiring general and reproductive health knowledge for exercising reproductive autonomy and agency [59,60]. Schooling and education also protect against unplanned pregnancy and early marriage [33,34] and empower AGYW with information for making safer reproductive health choices, such as condom use [62] and intention to [63] and actual utilisation of modern contraceptives [64]. Consequently, government and development partners must continue to expand and increase universal access to high-quality education, including reproductive health education, for AGYW in SSA.

Employment is another pathway through which education plays a protective role against adverse reproductive health outcomes. Educated women are more likely to be in employment than women who are not educated. We found that AGYW in employment reported a higher level of modern contraceptive use, even though they are also more likely to report becoming primiparous before 18 years. This complexity could be attributable to some factors. For instance, higher use of modern contraceptives could be directly related to career-induced need to plan childbirth or the possession of social capital to access and utilise reproductive health information. On the flip side, the possibility of becoming primiparous before 18 years may also have implications for policies that not only encourage the participation of women in the workforce but also guarantee and enforce their reproductive health and rights.

Just as in employment, we also found mixed results in the association between media exposure and reproductive health outcomes. We found that regular access to newspapers, radio and television increased the likelihood of reporting the use of modern contraceptives. This result shows the significance of information access on AGYW reproductive health and rights as reported in previous studies [3,7,32,61]. On the other hand, a higher likelihood to report early sexual debut and the likelihood of becoming primiparous before the age of 18 signal a need for not just regulating media content but also empowering AGYW to consume media content with a higher level of criticality and discretion. The mixed results in early sexual debut and early pregnancy/childbirth imply that media communications ought to be contextually fitted to respond to the current cultural storyline, stigma, and misrepresentation about youthful sexuality.

As expected, we found notable urban-rural disparities as AGYW in rural setting were less likely to be using modern contraceptives and more likely to report early sexual initiation and becoming primiparous before 18 years. This disparity reflects structural inequalities and inequities, such as poorer health systems and poverty, and stronger conservative norms [6,65] especially in relation to apathy towards the use of modern contraceptives. These structural inequalities could intersect with water insecurity to further compound vulnerability to adverse reproductive health outcomes. Therefore, it is imperative to prioritise integrated rural development strategies, including water, health and sanitation as well as sexual and reproductive health and rights actions in rural areas.

### Strengths and limitations

The study has strengths and some limitations that should be considered in evaluating the results and conclusions. First, we draw from a large and representative sample, implying that our results and conclusions are very likely to represent the SSA region. Second, we use data from the highly rated DHS data set, which is globally recognised as high-quality data collected through rigorous and standardized protocols. Finally, we used advanced and sophisticated statistical techniques to analyse data. The import of all these is that sourcing representative and large volumes of data from a high-quality and robust dataset and using advanced statistical techniques for analysis strengthens the quality, validity and reliability of our results and conclusions.

These strengths notwithstanding, our results and conclusions still need to be understood within some caveats. The self-reporting nature of the DHS opens up the possibility of underreporting or even social desirability bias. Early sexual debut, contraceptive use, and even becoming primiparous before 18 years could be seen as socially stigmatized, and this may impact reporting. Also, the lack of country-specific and place-specific analysis, even within countries, may also impact tailored interventions. Furthermore, health insurance was treated as a binary variable (insured vs. uninsured), as the DHS does not provide information on coverage extent or comprehensiveness, which may limit the interpretation of differences in financial protection. Moreover, wealth was measured using the standard DHS quintiles (poorest, poorer, middle, richer, richest). While this allows for cross-country comparability, it may obscure meaningful differences within categories, and the relative nature of wealth across countries may limit the interpretation of socioeconomic effects. Despite these limitations, we believe that our study opens up an important aspect of scholarship and provides background for future studies. We also believe that our results and conclusions could trigger conversations and strategic actions to address water insecurity and other social stressors that predispose AGYW in SSA to adverse reproductive health outcomes.

## Conclusion

The result of our study makes it imperative to prioritise and address water insecurity and other intersecting social and environmental stressors predisposing AGYW in SSA to adverse sexual and reproductive health outcomes. Adolescents aged 15–19 years are particularly more vulnerable, requiring equitable strategies to reach this group. Interventions to improve reproductive health and rights for AGYW in SSA must consider multi-faceted efforts that address intersecting vulnerabilities such as younger age, living in a rural area, having low levels or no formal education and experiencing poverty.
